# Incidence of extended-spectrum beta-lactamase-producing Klebsiella pneumoniae in patients with urinary tract infection

**DOI:** 10.1590/S1516-31802012000100007

**Published:** 2012-02-13

**Authors:** Sobhan Ghafourian, Zamberi Sekawi, Vasanthakumari Neela, Afra Khosravi, Mohammad Rahbar, Nourkhoda Sadeghifard

**Affiliations:** I MSc. Clinical Microbiology Research Center, Ilam University of Medical Sciences,Ilam, Iran. Department of Medical Microbiology & Parasitology, Faculty of Medicine and Health Sciences, Universiti Putra Malaysia, Selangor, Malaysia.; II MD, PhD. Associate Professor, Department of Medical Microbiology & Parasitology, Faculty of Medicine and Health Sciences, Universiti Putra Malaysia, Selangor, Malaysia.; III PhD. Assistant Professor, Department of Medical Microbiology & Parasitology, Faculty of Medicine and Health Sciences, Universiti Putra Malaysia, Selangor, Malaysia.; IV PhD. Associate Professor, Clinical Microbiology Research Center, Ilam University of Medical Sciences, Ilam, Iran.; V PhD. Associate Professor, Department of Microbiology, Iranian Reference Health Laboratories, Tehran, Iran.

**Keywords:** Klebsiella pneumoniae, Beta-lactamases, Urinary tract infections, Cephalosporins, Iran, Klebsiella pneumoniae, Beta-lactamases, Infecções urinárias, Cefalosporinas, Irã (geográfico)

## Abstract

**CONTEXT AND OBJECTIVES:**

Resistant bacteria are emerging worldwide as a threat to favorable outcomes from treating common infections in community and hospital settings. The present investigation was carried out to study the incidence of extended-spectrum beta-lactamase (ESBL)-producing *Klebsiella pneumoniae* in patients with urinary tract infection in different seasons of the year, in order to determine the prevalence of the genes blaTEM, blaSHV and blaCTX-M, which are responsible for ESBL production among ESBL-producing *K. pneumoniae*, in three cities in Iran, and to investigate the antimicrobial susceptibility pattern of *K. pneumoniae* in different seasons.

**DESIGN AND SETTING:**

Retrospective study carried out among patients with urinary tract infections in five hospitals in Iran.

**METHOD:**

Two hundred and eighty-eight clinical isolates of *K. pneumoniae* were collected between March 2007 and April 2008 from five hospitals in three cities in Iran. ESBLs were identified by phenotypic and genotypic methods. ESBL-producing *Klebsiella pneumoniae* were evaluated against non-beta-lactam antibiotics. Genes coding for ESBLs (blaSHV, TEM and CTX-M) were screened.

RESULTS: Among the 288 clinical isolates of *K. pneumoniae*, 37.7%, 46.7% and 15.6% were obtained from hospitals in Ilam, Tehran and Tabriz, respectively, of which 39.4%, 50.7% and 45.8% were ESBL-producing *K. pneumoniae* in Ilam, Milad and Emam Reza hospitals, respectively.

**CONCLUSION:**

According to the results from this study, resistance to third-generation cephalosporins is higher during the cold months than during the warm months.

## RESUMO

**CONTEXTO E OBJETIVOS:**

As bactérias resistentes estão surgindo em todo o mundo como uma ameaça ao resultado favorável no tratamento de infecções comuns em ambientes comunitários e hospitalares. O presente estudo foi realizado com o objetivo de avaliar a incidência de *Klebsiella pneumoniae* produtoras de beta-lactamases de espectro estendido (BLEEs) em pacientes com infecção do trato urinário em diferentes estações do ano, para verificar a prevalência dos genes blaTEM, blaSHV e blaCTX-M responsáveis para a produção de BLEEs em*K. pneumoniae* productoras de BLEEs, em três cidades do Irã, e investigar o perfil de sensibilidade antimicrobiana de *K. pneumoniae* em diferentes estações.

**DESENHO E LOCAL:**

Estudo retrospectivo realizado em pacientes com infecções do trato urinário em cinco hospitais no Irã.

**MÉTODO:**

Duzentos e oitenta e oito isolados clínicos de *K. penumoniae* foram coletados entre março de 2007 e abril de 2008 em cinco hospitais de três cidades no Irã. BLEEs foram identificados por métodos fenotípicos e genotípicos. *K. pneumoniae* produtoras de BLEEs foram avaliadas contra antibióticos não beta-lactâmicos. Os genes codificadores de BLEEs (blaSHV, TEM e CTX-M) foram investigados.

**RESULTADOS:**

Entre os 288 isolados clínicos de *K. pneumoniae*, 37,7%, 46,7% e 15,6% eram provenientes dos hospitais em Ilam, Tehran e Tabriz, respectivamente, dos quais 39,4%, 50,7% e 45,8% eram *K. pneumoniae* produtoras de BLEEs nos hospitais em Ilam, Milad e Eman Reza, respectivamente.

**CONCLUSÃO:**

De acordo com os resultados deste estudo, a resistência às cefalosporinas de terceira geração é maior nos meses frios do que nos meses quentes.

## INTRODUCTION

Resistant bacteria are emerging worldwide as a threat to beneficial outcomes from treating common infections in community and hospital settings.^1^ The introduction of third-generation cephalosporins into clinical practice in the early 1980s was heralded as a major breakthrough in the fight against beta-lactamase-mediated bacterial resistance to antibiotics. These cephalosporins had been developed in response to the increased prevalence of beta-lactamases in certain organisms (e.g. ampicillin-hydrolyzing TEM-1 and SHV-1 beta-lactamases in *Escherichia coli* and *Klebsiella pneumoniae*), as well as in response to the spread of such beta-lactamases into new hosts (e.g. *Haemophilus influenzae* and *Neisseria gonorrhoeae*). The third-generation cephalosporins were not only effective against most beta-lactamase-producing organisms, but also had the advantage of lower nephrotoxic effects than shown by aminoglycosides and polymyxins. However, plasmid-encoded beta-lactamases capable of hydrolyzing the extended-spectrum cephalosporins were soon reported, in 1983.^2^

Extended-spectrum beta-lactamases (ESBLs) are known as extended-spectrum because they are able to hydrolyze a broader spectrum of beta-lactam antibiotics than the simple parent beta-lactamases from which they are derived. Such ESBLs also have the ability to inactivate beta-lactam antibiotics containing an oxyimino group, such as oxyimino-cephalosporins (e.g. ceftazidime, ceftriaxone or cefotaxime) or oxyimino-monobactam.^3^ Furthermore, they are not active against cephamycins and carbapenems. Generally, they are inhibited by beta-lactamase inhibitors such as clavulanate and tazobactam. ESBLs have been found in a wide range of Gram-negative rods. However, the vast majority of strains expressing these enzymes belong to the *Enterobacteriaceae* family.^4^*K. pneumoniae* and *E. coli* stand out as the major ESBL producers.^5^

Strong selective pressure due to the use of beta-lactam drugs has resulted in the emergence of ESBL-producing strains. Expression of different types of ESBLs and selection of complex mutant enzymes with inhibitor-resistant phenotypes or porin shifts has also led to the development of resistance to cephamycins and other antimicrobials.^6^^,^^7^^,^^8^ The plasmids that harbor genes encoding ESBLs frequently contain other genes encoding mechanisms of resistance to aminoglycoside and cotrimoxazole.^9^ ESBLs are clinically important because they destroy cephalosporins, the workhorse hospital antibiotics that are given as first-line agents to many severely ill patients. Delayed recognition and inappropriate treatment of severe infections caused by ESBL producers acting on cephalosporins have been associated with increased mortality.^10^ It is important to study the prevalence of *K. pneumoniae* in Iran, since it is a country with four seasons.

## OBJECTIVE

This study was carried out to evaluate the incidence of extended-spectrum beta-lactamase-producing *K. pneumoniae* in patients with urinary tract infection in different seasons, in order to determine the prevalence of the genes blaTEM, blaSHV and blaCTX-M, which are responsible for ESBL production among ESBL-producing *K. pneumoniae*, in three cities in Iran, and to investigate the antimicrobial susceptibility pattern of *K. pneumoniae* in different seasons.

## METHODS

### Bacterial isolates

Two hundred and eighty-eight clinical isolates of *K. pneumoniae* associated with urinary tract infections, which were isolated in five hospitals in three cities in Iran between March 2007 and April 2008, were studied. The cities and hospitals included in this study were: Ilam (Emam Khomaini, Mostafa Khomaini and Ghaem Hospitals), Tabriz (Emam Reza Hospital) and Tehran (Milad Hospital). 

### Sample collection

A volume of urine measured using the calibrated loop method was inoculated into nutrient agar medium for colony counting. Densities of single-potential *K. pneumoniae* greater than or equal to 10^4^ CFU/ml (colony forming units per milliliter) were interpreted as positive for urinary tract infection, while the test was repeated if the result was 10^2^-10^4^ CFU/ml. Results less than 10^2^ CFU/ml were interpreted as negative for urinary tract infection.^11^ Urine specimens were cultured for isolation of *K. pneumoniae* in blood agar and MacConkey agar media.

### Identification of K. pneumoniae

All isolates were subjected to standard confirmatory tests, which included Gram staining, oxidase and catalase, growth on SIM (sulfide - indole - Motility), Simon citrate, MR-VP (Methyl Red - Voges Proskauer), lysine iron agar, Kligler agar, phenylalanine agar, urea agar, malonate, Blood Agar, MacConkey agar.^12^

### ESBL detection

ESBL production was detected by using the disk diffusion method. In keeping with the Clinical and Laboratory Standards Institute (CLSI) recommended guidelines, ESBL screening was performed by means of disk diffusion using ceftazidime (30 µg), cefotaxime (30 µg), ceftriaxone (30 µg), aztreonam (30 µg) and cefpodoxime (30 µg) disks. The ESBL phenotype was confirmed by means of the double disk diffusion method, using antibiotic disks containing a combination of cephalosporin plus clavulanic acid, in conjunction with the corresponding cephalosporin disk alone. The following antibiotic disks were used: ceftazidime (30 µg); ceftazidime plus clavulanic acid (30/10 µg); cefotaxime (30 µg); cefotaxime plus clavulanic acid (30/10 µg); and cefpodoxime (30 µg) plus clavulanic acid (30/10 µg). The tests were interpreted in accordance with the CLSI guidelines. Regardless of the zone diameters, an increase in zone diameter > 5 mm for an antimicrobial agent tested in combination with clavulanic acid, in comparison with its zone size when tested alone, indicated probable ESBL production.^13^*K. pneumoniae* ATCC 700603 was used as a control for the ESBL tests. The antibiotic susceptibility pattern of the ESBL-producing isolates was recorded by means of the disk diffusion method, using the following antimicrobial agents: amikacin (30 µg), ciprofloxacin (30 µg), cotrimoxazole (30 µg) and imipenem (30 µg).^14^

### Polymerase chain reaction amplification of blaTEM, blaSHV and blaCTX-M genes

The polymerase chain reaction was carried out using the following primers: blaTEM (forward 5’-GAGTATCAACATTTCCGTGTC-3’, reverse 5’-TAATCAGTGAGGCACCTTCTC-3’); blaSHV (forward 5’-AAGATCCACTATCGCCCAGCAG-3’, reverse 5’-AAGATCCACTATCGC CCAGCAG-3’);^15^ and blaCTX-M (forward 5’-ACGCTGTTGTTAGGAAGTG-3’, reverse 5’-TTGAGGCTGGGTGAAGT-3’).^16^

The blaSHV gene was amplified under the following conditions: initial denaturation at 94 °C for 3 min, followed by 35 cycles of denaturation at 95 °C for 30 seconds, annealing at 56 °C for 1 min, and 72 °C for 1 min, with a final extension at 72 °C for 10 min. The blaTEM gene was amplified under the following conditions: initial denaturation at 94 °C for 3 min, followed by 35 cycles of denaturation at 95 °C for 30 seconds, annealing at 45 °C for 1 min, and 72 °C for 1 min, with a final extension at 72 °C for 10 min. The blaCTX-M gene was amplified under the following conditions: initial denaturation at 94 °C for 3 min, followed by 35 cycles of denaturation at 95 °C for 30 seconds annealing at 48 °C for 1 min, and 72 °C for 1 min, with a final extension at 72 °C for 10 min. The amplicons were run on 1% agarose gel. The gels were stained with ethidium bromide, and bands observed at the desired position were photographed using an ultraviolet light transilluminator.

The statistical analyses to determine the frequency of antimicrobial resistance were descriptive and performed using the Statistical Package for the Social Sciences (SPSS), version 13.

## RESULTS

Among the 288 clinical isolates of *K. pneumoniae* obtained from five hospitals in three cities in Iran, 37.7% (n = 109), 46.7% (n = 134) and 15.6% (n = 45) were isolated from hospitals in Ilam, Tabriz and Tehran, respectively, of which 39.4% (n = 43), 50.7% (n = 68) and 45.8% of *K. pneumoniae* were ESBLs positive in hospitals in Ilam, Tehran and Tabriz, respectively. 

Out of the total number of *K. pneumoniae* isolates available for this study, 18.75% (n = 54), 12.84% (n = 37), 33.3% (n = 96) and 35.1% (n = 101) were isolated in the spring, summer, fall and winter, respectively. The number of *K. pneumoniae* isolates in different seasons (spring, summer, fall and winter) from different hospitals and the corresponding ESBL patterns are presented in Table 1, while the antibiotic susceptibility pattern towards other antibiotic panels is shown in Table 2. The numbers of *K. pneumoniae* isolates were found to be high in the fall and winter seasons.

### Polymerase chain reaction results

**Figure 1.**
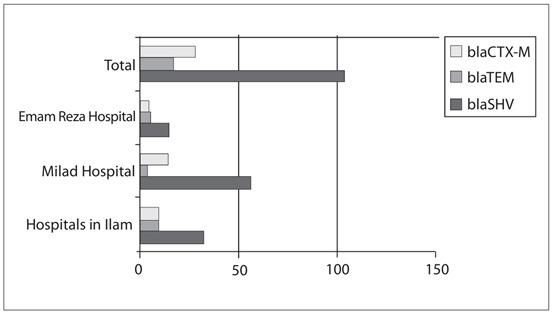
Frequencies of blaSHV, blaTEM and blaCTX-M in different hospitals.

The frequencies of ESBL genes varied among the different hospitals in different seasons. In total, 104, 22 and 17 isolates were positive for blaSHV, blaCTX-M and blaTEM genes, respectively. In the hospitals in Ilam, out of the eight *K. pneumoniae* isolates with blaTEM, 12.5% (n = 1), 12.5% (n = 1), 25% (n = 2) and 50% (n = 4) were obtained in the spring, summer, fall and winter, respectively. Out of the 32 *K. pneumoniae* isolates with blaSHV, 21.9% (n = 7), 6.25% (n = 2), 28.1% (n = 9) and 43.75% (n = 14) were found in the spring, summer, fall and winter, respectively. For *K. pneumoniae* with blaCTX-M, 20% (n = 2), 10% (n = 1), 20% (n = 2) and 50% (n = 5) were acquired in the spring, summer, fall and winter, respectively. Among the patients with urinary tract infections in Milad Hospital (in Tehran), 57 *K. pneumoniae* isolates with blaSHV were observed. Of these, 28.1% (n = 16), 8.7% (n = 5), 28.1% (n = 16) and 35.1% (n = 20) were found in the spring, summer, fall and winter, respectively. Out of the four *K. pneumoniae* isolates with blaTEM, 25% (n = 1), 25% (n = 1) and 50% (n = 2) were found in the spring, fall and winter, respectively. Our findings also showed that, among the 14 *K. pneumoniae* isolates with blaCTX-M from the patients with urinary tract infections, 7.1% (n = 1) were obtained in the spring, 21.5% (n = 3) in the fall and 71.4% (n = 10) in the winter. In Emam Reza Hospital (in Tabriz), among the 15 *K. pneumoniae* isolates with blaSHV from patients with urinary tract infections, 100% (n = 1), 88.9% (n = 8) and 55.4% (n = 6) were found in the spring, fall and winter, respectively. Five *K. pneumoniae* isolates with blaTEM were observed, of which 22.3% (n = 2) were obtained in the fall and 27.3% (n = 3) in the winter. Out of the four *K. pneumoniae* isolates with blaCTX-M from the patients with urinary tract infections, 11.1% (n = 1) and 27.3% (n = 3) acquired it in the fall and winter, respectively (Figure 1).

**Table 1.** Incidence of *Klebsiella pneumoniae* and antimicrobial susceptibility patterns for ESBL detection in different seasons

Hospitals	Seasons	*K. pneumoniae*	Ceftazidime	Cefotaxime	Ceftriaxone	Cefpodoxime	Aztreonam
Hospitals in Ilam	Spring	18 (16.52%)	6 (33.33%)	12 (66.66%)	9 (50%)	7 (38.88%)	7 (38.88%)
	Summer	14 (12.85%)	2 (14.28%)	3 (21.42%)	6 (42.85%)	3 (21.42%)	3 (21.42%)
	Fall	37 (33.94%)	15 (40.54%)	18 (48.64%)	14 (37.83%)	12 (32.43%)	12 (32.43%)
	Winter	40 (36.69%)	25 (62.5%)	22 (55%)	31 (77.5%)	22 (55%)	21 (52.5%)
	Total	109 (100%)	48 (44%)	55 (50.45%)	60 (55%)	44 (40.3%)	43 (39.4%)
Milad Hospital	Spring	31 (23.14%)	11 (35.48%)	19 (61.29%)	18 (58.06%)	16 (51.61%)	16 (51.61%)
	Summer	21 (15.67%)	9 (42.85%)	2 (9.52%)	8 (38.09%)	5 (23.8%)	5 (23.8%)
	Fall	39 (29.1%)	21 (53.85%)	15 (38.46%)	19 (48.71%)	18 (46.15%)	18 (46.15%)
	Winter	43 (32.08%)	32 (74.41%)	18 (41.86%)	32 (74.41%)	29 (67.44%)	29 (67.44%)
	Total	134 (100%)	73 (54.4%)	54 (40.3%)	77 (57.5%)	68 (50.74%)	68 (50.74%)
Emam Reza Hospital	Spring	5 (11.11%)	2 (40%)	0	2 (40%)	1 (20%)	1 (20%)
	Summer	2 (4.44%)	1 (50%)	0	0	0	0
	Fall	20 (44.44%)	8 (40%)	11 (55%)	10 (50%)	9 (45%)	9 (45%)
	Winter	18 (40%)	14 (77.77%)	9 (50%)	16 (88.88%)	11 (61.11%)	11 (61.11%)
	Total	45 (100%)	25 (55.5%)	20 (44.4%)	28 (62.2%)	21 (46.6%)	21 (46.6%)
Total	Spring	54 (18.75%)	19 (35.1%)	31 (57.4%)	29 (53.7%)	24 (44.4%)	24 (44.4%)
	Summer	37 (12.84%)	12 (32.4%)	5 (13.5%)	14 (37.8%)	8 (21.6%)	8 (21.6%)
	Fall	96 (33.3%)	44 (45.8%)	44 (45.8%)	43 (44.8%)	39 (40.6%)	39 (40.6%)
	Winter	101 (35.1%)	71 (70.3%)	49 (48.5%)	79 (78.2%)	62 (61.4%)	61 (60.4%)
	Total	288 (100%)	146 (50.7%)	129 (44.8%)	165 (57.3%)	133 (46.2%)	132 (45.8%)

ESBL = extended-spectrum beta-lactamase.

**Table 2.** Antimicrobial susceptibility testing on ESBL-producing Klebsiella pneumoniae in different seasons

		***K. pneumoniae* positive for ESBLs**	Amikacin	Ciprofloxacin	Cotrimoxazole	Imipenem
Hospitals in Ilam	Spring	7 (16.3%)	0	0	0	0
	Summer	3 (7%)	0	0	0	0
	Fall	12 (27.9%)	1 (8.3%)	0	0	0
	Winter	21 (48.8%)	2 (9.52%)	1 (4.76%)	1 (4.76%)	0
	Total	43 (100%)	3 (9.6%)	1 (2.3%)	1 (2.3%)	0
Milad Hospital	Spring	16 (23.5%)	5 (31.25%)	4 (25%)	6 (37.5%)	0
	Summer	5 (7.35%)	2 (40%)	1 (20%)	2 (40%)	0
	Fall	18 (26.4%)	6 (33.3%)	5 (27.8%)	9 (50%)	0
	Winter	29 (42.65%)	12 (41.4%)	6 (20.7%)	9 (31%)	0
	Total	68 (100%)	25 (36.7%)	16 (23.5%)	26 (38.2%)	0
Emam Reza Hospital	Spring	1 (4.8%)	0	0	0	0
	Fall	9 (42.8%)	3 (33.3%)	0	2 (22.2%)	0
	Winter	11 (52.4%)	4 (36.4%)	1 (9%)	2 (18.8%)	0
	Total	21 (100%)	7 (33.3%)	1 (4.8%)	4 (19%)	0
Total	Spring	24 (18.2%)	5 (20.8%)	4 (16.67%)	6 (25%)	0
	Summer	8 (6%)	2 (25%)	1 (12.5%)	2 (25%)	0
	Fall	39 (29.6%)	10 (25.6%)	5 (12.8%)	11 (28.2%)	0
	Winter	61 (46.2%)	18 (29.5%)	8 (13.1%)	12 (19.7%)	0
	Total	132 (100%)	35 (26.5%)	18 (13.6%)	31 (23.5%)	0

ESBL = extended-spectrum beta-lactamase.

## DISCUSSION

The development of extended-spectrum cephalosporins in the early 1980s was regarded as a major addition to the therapeutic armamentarium in the fight against beta-lactamase-mediated bacterial resistance.^17^ Regrettably, the emergence of *K. pneumoniae* presenting resistance to ceftazidime and other cephalosporins has seriously compromised the efficacy of these life-saving antibiotics.

The data obtained from clinical samples of *K. pneumoniae* now show high antibiotic resistance. The resistance to antibiotics shown by *Klebsiella* has increased worldwide.^18^ Carbapenems are the drugs of choice for many infections caused by Gram-positive and Gram-negative bacteria.^19^ Imipenem has been found to be the most effective antibiotic. Our results indicated that all isolates were susceptible to imipenem. This is consistent with the findings of Alzahrani and Akhtar from Saudi Arabia.^20^ Gram-negative pathogens harboring ESBLs have caused numerous outbreaks of infections and are becoming an increasing therapeutic problem in many countries. The incidence of ESBL-producing strains among clinical isolates has been steadily increasing over the past years, thus resulting in limitations to the therapeutic options.^21^ ESBLs are now a significant problem in hospitalized patients throughout the world. The prevalence of ESBLs among clinical isolates varies worldwide, and the patterns are rapidly changing over time.^22^ Patients suffering from infections caused by ESBL-producing organisms are at higher risk of treatment failure when broad-spectrum beta-lactam antibiotics are used. Therefore, it is recommended that any organisms experimentally confirmed as ESBL producers should be reported as resistant to the entire broad-spectrum beta-lactam antibiotic, regardless of any susceptibility test results.^14^ The phenotypic test only presumptively identifies the presence of ESBL. The task of identifying the specific ESBLs present in clinical isolates is more complicated. The easiest and most common molecular method used to detect the presence of a beta-lactamase belonging to a family of enzymes is the polymerase chain reaction, using oligonucleotide primers that are specific for the beta-lactamase gene. These primers are usually chosen to anneal to regions where various point mutations are not known to occur.^23^

The current study was conducted during different seasons and in hospitals in different parts of Iran. To the best of our knowledge, this study was the first to cover different seasons and hospitals all at once. In the hospitals in Ilam, comparison between different seasons revealed that the highest resistance to third-generation cephalosporins among patients with urinary tract infections was for ceftriaxone in the winter (77.5%). The results showed that ESBL production by *K. pneumoniae* was higher in winter than in the other seasons. Interestingly, similar results recurred for ciprofloxacin, cotrimoxazole and amikacin. The dominant gene responsible for ESBL production was blaSHV (n = 32) and the lowest frequency observed was for blaTEM (n = 8). As expected, the highest frequency of these three genes responsible for ESBL production was observed in the winter. Ceftazidime and ceftriaxone resistance was highest in patients with urinary tract infections at the screening stage. In these patients, the highest levels of ESBL-producing *K. pneumoniae* (42.65%) and amikacin resistance were found in winter (41.4%) while resistance to ciprofloxacin (27.8%) and cotrimoxazole (50%) was greater in the fall than in the other seasons. The results reflected the observation that blaSHV was the dominant gene responsible for ESBL production, followed by blaSHV and blaCTX-M. The frequencies of all three genes in the winter were more than in the other seasons, while the lowest frequency was in the summer. Our findings demonstrated that *K. pneumoniae* presented greater resistance to antibiotics and higher ESBL production during the cold seasons than during the warm seasons. 

In patients with urinary tract infections in Emam Reza Hospital, the highest resistance to third-generation cephalosporins was found for ceftriaxone (88.8%) in the winter. The ESBL production by *K. pneumoniae* was 52.4%, while resistance to amikacin was 36.4% and to ciprofloxacin, 9%, in the winter. Resistance to cotrimoxazole was 22.2% in the fall, which was higher than in the other seasons. 

Climatic factors may also be important in the pathogenesis of urinary tract infections. There have been several reports suggesting that there is higher incidence of such infections in cold weather. Most studies suggest that these infections are particularly likely to occur when patients dress in a manner inappropriate to the weather conditions.^24^ Our results showed variations in the incidence rate throughout the year, with a decrease in warm seasons. One possible explanation, suggested by Stansfeld, is that upper respiratory tract infections may precede urinary tract infections. Since coughs and colds are more common in the winter months, the same would apply to urinary tract infections.^25^

In a study in Milad Hospital in Tehran carried out between March and June 2009, out of 115 strains of *K. pneumoniae* from urine specimens, 12% of the *K. pneumoniae* isolates were found to be positive for ESBLs.^26^ This showed a decline in ESBL production in urinary tract infections, compared with our findings from Milad Hospital one year earlier (50.7%). In another investigation on the prevalence and antimicrobial resistance patterns at a tertiary care hospital in northern India, out of the 100 isolates from different *Klebsiella* species, 56% were ESBL producers. About 85% of the ESBL producers were resistant to cephalosporins. All the isolates were sensitive to imipenem.^27^ These results were in accordance with our study.

One hundred and sixty-eight clinical isolates of *K. pneumoniae* were collected in a survey between September 2006 and February 2007, from three general hospitals in Tehran, Iran. It was found that 69% were positive for ESBLs.^28^ This finding demonstrated lower frequency of ESBL production, in comparison with the study by Bameri in Milad Hospital, Emam Reza Hospital and hospitals in Ilam, in Iran. However, the frequency in the three hospitals in Tehran was over twice the frequency found by Irajian et al. in Semnan, Iran, with 28.9% ESBL production.^11^

In Saudi Arabia in 2007, out of 400 *K. pneumoniae* isolates investigated, 55% were positive for ESBLs, 97.3% for blaSHV and 84.1% for blaTEM genes. The resistance rates to cefotaxime and ceftazidime were 97% and 95%, respectively. All the ESBL-producing isolates were susceptible to imipenem.^29^ The results from Milad Hospital, Emam Reza Hospital and the hospitals in Ilam also showed high frequency of blaSHV, while the frequency of blaTEM was lower than in the study done in Saudi Arabia. Our study showed that imipenem was an effective antibiotic for *K. pneumoniae*, which is in agreement with Al-Agamy et al. in 2007 in Saudi Arabia.^29^

In Dokuz Eylul University Hospital in Turkey, 38% of *Enterobacteria* isolates were ESBL positive.^30^ A similar result was found in hospitals in Ilam (39.4% positive for ESBLs) while in Milad and Emam Reza Hospitals, the frequency of ESBL-producing *K. pneumoniae* was higher than in Dokuz Eylul University Hospital. In the latter, 52.7% showed blaTEM and 74.3% blaSHV, while the high frequency of blaSHV and low frequency of blaTEM were again found in Milad and Emam Reza Hospitals and the hospitals in Ilam. 

## CONCLUSION

According to the results from this study, resistance to third-generation cephalosporins was generally higher during the cold months than during the warm months. Furthermore, resistance to cefpodoxime was almost equal to that of aztreonam, while resistance to other cephalosporins used in this study varied considerably.

Our study showed that *K. pneumoniae* recovered from urine specimens produced a higher number of ESBLs. It also showed resistance towards fluoroquinolones, aminoglycosides and cotrimoxazole.

The gene predominantly responsible for ESBL production was blaSHV. The levels of ESBLs produced in Milad Hospital, in Tehran, were much higher than those in other hospitals covered in this study. In all the hospitals, imipenem was used as an effective antibiotic against ESBL-producing *K. pneumoniae*. Resistance to ciprofloxacin, which was commonly seen in the cold seasons, was low, but resistance to cotrimoxazole and amikacin was found to be almost the same but higher than the resistance to ciprofloxacin. Strict antibiotic policies should be adopted in hospitals, in order to estimate the impact of higher resistance among bacteria and to take steps towards reducing this resistance.

## References

[1] Chaudhary U, Aggarwal R (2004). Extended spectrum -lactamases (ESBL) - an emerging threat to clinical therapeutics. Indian J Med Microbiol.

[2] Knothe H, Shah P, Krcmery V, Antal M, Mitsuhashi S (1983). Transferable resistance to cefotaxime, cefoxitin, cefamandole and cefuroxime in clinical isolates of Klebsiella pneumoniae and Serratia marcescens. Infection.

[3] Bradford PA (2001). Extended-spectrum beta-lactamases in the 21st century: characterization, epidemiology, and detection of this important resistance threat. Clin Microbiol Rev.

[4] Paterson DL, Bonomo RA (2005). Extended-spectrum beta-lactamases: a clinical update. Clin Microbiol Rev.

[5] Winokur PL, Brueggemann A, DeSalvo DL (2000). Animal and human multidrug-resistant, cephalosporin-resistant salmonella isolates expressing a plasmid-mediated CMY-2 AmpC beta-lactamase. Antimicrob Agents Chemother.

[6] Bradford PA, Cherubin CE, Idemyor V, Rasmussen BA, Bush K (1994). Multiply resistant Klebsiella pneumoniae strains from two Chicago hospitals: identification of the extended-spectrum TEM-12 and TEM-10 ceftazidime-hydrolyzing beta-lactamases in a single isolates. Antimicrob Agents Chemother.

[7] Pangon B, Bizet C, Buré A (1989). In vivo selection of a cephamycin-resistant, porin-deficient mutant of Klebsiella pneumoniae producing a TEM-3 beta-lactamase. J Infect Dis.

[8] Sirot D (1995). Extended-spectrum plasmid-mediated beta-lactamases. J Antimicrob Chemother.

[9] Villa L, Pezzella C, Tosini F (2000). Multiple-antibiotic resistance mediated by structurally related IncL/M plasmids carrying an extended-spectrum beta-lactamase gene and a class 1 integron. Antimicrob Agents Chemother.

[10] Paterson DL, Ko WC, Von Gottberg A (2001). Outcome of cephalosporin treatment for serious infections due to apparently susceptible organisms producing extended-spectrum beta-lactamases: implications for the clinical microbiology laboratory. J Clin Microbiol.

[11] Irajian G, Moghadas AJ (2010). Frequency of extended-spectrum beta lactamase positive and multidrug resistance pattern in Gram-negative urinary isolates, Semnan, Iran. Jundishapur Journal of Microbiology.

[12] Mac Faddin JF (1999). Biochemical tests for identification of medical bacteria.

[13] Clinical and Laboratory Standards Institute (2010). Performance standards for antimicrobial susceptibility testing. Twentieth informational supplement, M100-S20.

[14] Paterson DL, Mulazimoglu L, Casellas JM (2000). Epidemiology of ciprofloxacin resistance and its relationship to extended-spectrum beta-lactamase production in Klebsiella pneumoniae isolates causing bacteremia. Clin Infect Dis.

[15] Shahcheraghi F, Moezi H, Feizabadi MM (2007). Distribution of TEM and SHV beta-lactamase genes among Klebsiella pneumoniae strains isolated from patients in Tehran. Med Sci Monit.

[16] Mansouri M, Ramazanzadeh R (2009). Spread of extended-spectrum beta-lactamase producing Escherichia coli clinical isolates in Sanandaj Hospitals. Journal of Biological Sciences.

[17] Bush K (2002). The impact of beta-lactamases on the development of novel antimicrobial agents. Curr Opin Investig Drugs.

[18] Shannon K, Phillips I (1986). The effects on beta-lactam susceptibility of phenotypic induction and genotypic derepression of beta-lactamase synthesis. J Antimicrob Chemother.

[19] Yu Y, Zhou W, Chen Y, Ding Y, Ma Y (2002). Epidemiological and antibiotic resistant study on extended-spectrum beta-lactamase-producing Escherichia coli and Klebsiella pneumoniae in Zhejiang Province. Chin Med J (Engl).

[20] Alzahrani AJ, Akhtar N (2005). Susceptibility patterns of extended spectrum beta-lactamase (ESBL)-producing Escherichia coli and Klebsiella pneumoniae isolated in a teaching hospital. Pakistan Journal of Medical Research.

[21] Podschun R, Ullmann C (1998). Klebsiella spp. as nosocomial pathogens: epidemiology, taxonomy, typing methods, and pathogenicity factors. Clin Microbiol Rev.

[22] Livermore DM (1995). beta-Lactamases in laboratory and clinical resistance. Clin Microbiol Rev.

[23] Arlet G, Philippon A (1991). Construction by polymerase chain reaction and use of intragenic DNA probes for three main types of transferable beta-lactamases (TEM, SHV, CARB). FEMS Microbiol Lett.

[24] Elo J, Sarna S, Tallgren LG (1979). Seasonal variations in the occurrence of urinary tract infections among children in an urban area in Finland. Ann Clin Res.

[25] Stansfeld JM (1966). Clinical observations relating to incidence and aetiology of urinary-tract infections in children. Be Med J.

[26] Behrooozi A, Rahbar M, Vand Yousefi J (2010). Frequency of extended spectrum beta-lactamase (ESBLs) producing Escherichia coli and Klebsiella pneumoniae isolated from urine in an Iranian 1000-bed tertiary care hospital. African Journal of Microbiology Research.

[27] Jain A, Mondal R (2007). Prevalence & antimicrobial resistance pattern of extended spectrum beta-lactamase producing Klebsiella spp isolated from cases of neonatal septicaemia. Indian J Med Res.

[28] Bameri Z, Chitsaz M, Owlia P (2010). Detection of CTX-M b - Lactamases in Isolated Klebsiella pneumoniae. Iranian Journal of Pathology.

[29] Al-Agamy MH, Shibl AM, Tawfik AF (2009). Prevalence and molecular characterization of extended-spectrum beta-lactamase-producing Klebsiella pneumoniae in Riyadh, Saudi Arabia. Ann Saudi Med.

[30] Tasli H, Bahar IH (2005). Molecular characterization of TEM- and SHV-derived extended-spectrum beta-lactamases in hospital-based Enterobacteriaceae in Turkey. Jpn J Infect Dis.

